# In Silico Peptide Ligation: Iterative Residue Docking and Linking as a New Approach to Predict Protein-Peptide Interactions

**DOI:** 10.3390/molecules24071351

**Published:** 2019-04-05

**Authors:** Julien Diharce, Mickaël Cueto, Massimiliano Beltramo, Vincent Aucagne, Pascal Bonnet

**Affiliations:** 1 Institut de Chimie Organique et Analytique (ICOA), UMR CNRS-Université d’Orléans 7311, Université d’Orléans BP 6759, 45067 Orléans Cedex 2, France; julien.diharce@univ-orleans.fr (J.D.); mickael.cueto@etu.univ-orleans.fr (M.C.); 2 UMR Physiologie de la Reproduction et des Comportements (INRA, UMR85; CNRS, UMR7247; Université de Tours; IFCE), F-37380 Nouzilly, France; massimiliano.beltramo@inra.fr; 3 Centre de Biophysique Moléculaire (CNRS UPR4301), Rue Charles Sadron, F-45071 Orléans Cedex 2, France; aucagne@cnrs-orleans.fr

**Keywords:** protein–peptide interaction, covalent docking, peptide conformation, molecular modeling

## Abstract

Peptide–protein interactions are corner-stones of living functions involved in essential mechanisms, such as cell signaling. Given the difficulty of obtaining direct experimental structural biology data, prediction of those interactions is of crucial interest for the rational development of new drugs, notably to fight diseases, such as cancer or Alzheimer’s disease. Because of the high flexibility of natural unconstrained linear peptides, prediction of their binding mode in a protein cavity remains challenging. Several theoretical approaches have been developed in the last decade to address this issue. Nevertheless, improvements are needed, such as the conformation prediction of peptide side-chains, which are dependent on peptide length and flexibility. Here, we present a novel in silico method, Iterative Residue Docking and Linking (IRDL), to efficiently predict peptide–protein interactions. In order to reduce the conformational space, this innovative method splits peptides into several short segments. Then, it uses the performance of intramolecular covalent docking to rebuild, sequentially, the complete peptide in the active site of its protein target. Once the peptide is constructed, a rescoring step is applied in order to correctly rank all IRDL solutions. Applied on a set of 11 crystallized peptide–protein complexes, the IRDL method shows promising results, since it is able to retrieve experimental binding conformations with a Root Mean Square Deviation (RMSD) below 2 Å in the top five ranked solutions. For some complexes, IRDL method outperforms two other docking protocols evaluated in this study. Hence, IRDL is a new tool that could be used in drug design projects to predict peptide–protein interactions.

## 1. Introduction

In biological systems, non-covalent interactions between two molecular entities, mostly receptor–ligand and protein–protein, are the corner-stones of cellular functions and communication. Their importance has been known for several decades and the study of the underpinning mechanisms is a very popular research field in biology and biochemistry. A better knowledge in this field would be of paramount importance in the design of new drugs targeting proteins involved in pathological conditions. To assist these research studies there has been a rapid growth of in silico approaches to predict molecular interactions. In particular, protein–protein interactions (PPIs) are an inherent phenomenon involved in key biological processes [1,2]. Amongst PPIs, the description of peptide–protein interactions is particularly important, since these interactions have a predominant role in many regulatory processes (e.g., neuropeptide–receptor and enzyme–substrate interactions), and may constitute between 15 and 40% of all interactions present in cells [3]. Characterization at the atomic level of the molecular interactions may provide key information to develop novel therapeutic treatments for cancer [4], neurodegenerative diseases [5], autoimmune diseases [6], and numerous other pathologies [7,8,9]. In addition, the design of modulating peptides, targeting mainly PPIs, becomes one attractive field of development in drug discovery [10,11,12,13,14,15]. By targeting the surface interaction of two bound proteins instead of their respective active site, the biological (or catalytic) activity of each protein could be maintained, while the downstream signaling processes could be modified [16]. However, elucidation by experimental methods of the structure of protein–peptide complexes still remains a challenge, due to the high flexibility of linear peptides [17,18] and their transient interactions. In addition, peptides may have a variable size, from a few residues to dozens of amino acids [19]. Consequently, the use of bioinformatics and in silico tools is a complementary approach that is increasingly applied to predict the structure of peptide–protein complexes [20].

Several molecular modeling methods and bioinformatics tools dedicated to the prediction of the structure of peptide–protein complexes have been developed in the past decade [18]. Methods such as PepATTRACT [21], PEP-FOLD3 [22], Rosetta FlexPepDock [23], HADDOCK [24], SP-peptide module of Glide [25], and others [26,27] have proven their capacity to predict peptide–protein complexes. Each tool has its own method to predict protein–peptide interactions, such as de novo prediction, coarse-grained resolution, restraint-guided prediction, etc. However, all these tools would benefit from refinement of the prediction of the sidechain position, especially when the considered peptide–protein interaction triggers a conformational change (e.g., receptor activation). Indeed, small modifications in the predicted binding of the backbone or the sidechain of the peptide could totally change the mode of interaction and may not trigger the activation of the biological target as observed experimentally. Another major difficulty to address is the strong induced fit of the peptide. After binding, the peptide could fold on the receptor surface, while it is unfolded in its unbound state [28]. Fragmentation of peptides is one of the methods to address the problem of peptide flexibility. Many in silico tools have already been developed to design ligands from fragments, such as LUDI [29], Dynamic Ligand Design (DLD) [30], or AutoGrow [31]. For the latter, the program is now able to predict drug-like properties and to optimize ligands with click chemistry rules [32]. However, those tools, which are used in several docking programs, such as Hammerhead [33] or FlexX [34], are not specifically developed for peptides, for which few tools currently exist. In the 1990’s, GROW was developed for the prediction of peptide–protein interactions, using single amino acids to construct peptides of interest within cavities [35]. Even if the approach brought, at that time, very useful insights into the design of peptidomimetics for the inhibition of HIV protease [36,37,38], it suffers from several drawbacks, such as the conformational sampling of the molecules in order to propose different solutions, or the prediction of the position of the starting seed.

In this current work, starting from the initial idea to consider peptide fragments instead of the whole peptides, we have designed a novel approach to predict peptide–protein interactions with the use of a covalent docking approach. The covalent docking protocol was initially developed to predict the covalent bond between an inhibitor and its biological target. We applied this concept to the prediction of protein–peptide interactions, but without creating a covalent bond between the peptide and the protein. Covalent docking is used here to iteratively build the peptide from several segments inside the binding site of a given receptor, which consists of a novel methodology employing this method. Our new approach has been compared to two docking modules of Glide [39], from the Schrödinger Suite, Standard Precision (SP), and SP-Peptide. Results show that in all cases "Iterative Covalent Docking and Linking" (IRDL) is able to retrieve the crystallographic pose of peptides. Furthermore, in 10 out of 11 cases, in the top 5 poses, IRDL is able to retrieve a Root-Mean Square Deviation (RMSD) below 2 Å between the docking pose and the crystal structure.

## 2. Results and Discussion

Protein peptide interaction is a crucial phenomenon in the living world and is involved in many biological processes. Here, we propose a docking approach to predict the correct molecular interactions and conformations of small unconstrained peptides bound to protein targets. To achieve this goal, exploration of the conformational space of the peptides should be exhaustive. However, classical searching algorithms show their limits when the number of rotatable bonds increases. Thus, the prediction of protein–peptide interaction, at atomic scale precision, becomes challenging, due to the high flexibility of these molecules. Specific conformational search tools have been developed to assess the binding mode of molecules with large numbers of rotatable bonds. We present here the development of a novel method that is able to retrieve the binding mode of peptides bound to proteins. Furthermore, we compare the results obtained with this model to those obtained by two classical docking protocols present in the same Glide software: standard precision (SP) protocol and SP-peptide protocol, developed specifically for protein–peptide interactions. All docking poses were rescored with the Extra Precision (XP) scoring function.

After rescoring with the XP scoring function, we selected all docking poses from the highest scoring pose to the pose with the lowest RMSD to the X-ray structure. We also took into account the position of reasonable docking poses, meaning poses with a deviation lower than 2 Å and 3 Å, considering the backbone or the whole peptide, respectively. For analysis, RMSD calculation was performed without hydrogens. In general, the crystal structure of the peptides with a RMSD lower than 2 Å, considering the whole peptide compared to the docking pose, was found in at least one protocol for all case studies, but did not match with the top scoring position. This is observed with many docking tools, where the top solution is often not the one with the lowest RMSD to crystal structure.

### 2.1. Highest Scoring Pose

**Figure 1:**
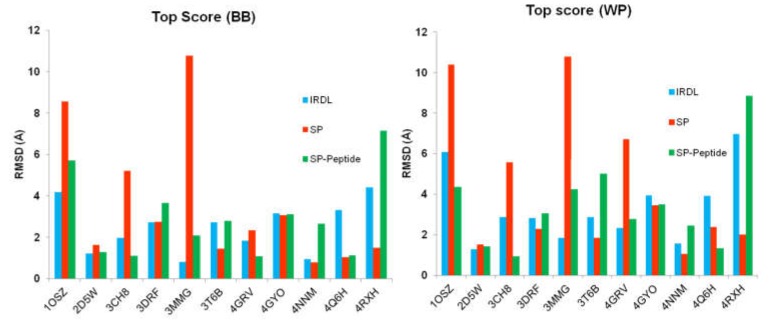
Root Mean Squared Deviation (RMSD) values obtained for the top scoring position after rescoring with the Extra Precision (XP) scoring function for the three protocols Iterative Residue Docking and Linking (IRDL), Standard Precision (SP), and SP-Peptide. BB: only the peptide backbone is considered; WP: the whole peptide is taken into account. RMSD values are given in Ångstrom.

The top scoring solution of each methodology was obtained by ranking all poses with Glide XP scoring function. In addition, we also analyzed the rank of the first pose having a root-mean square deviation (RMSD) below 2 Å and 3 Å for the backbone and the whole peptide, respectively. Figure 1 represents the RMSD of the top scoring pose, after rescoring all poses with Glide XP scoring function, compared to the X-ray structure of each peptide using the three protocols. Details of all calculated RMSD values of the top scoring position are given in Table S3.

Taking into account only the top scoring solution, the results show good RMSD values for some peptide–protein complexes but are unreasonable for some other examples, regardless of the applied protocols. SP protocol fails to retrieve a correct solution for four complexes (PDB ID 1OSZ, 3CH8, 3MMG, and 4GRV) with a RMSD below 2 or 3 Å when considering the backbone or the whole peptide, respectively, but it can predict a solution very close to the X-ray structure, such as for PDB ID 4NNM. This classical docking protocol developed for protein-ligand complexes has shown high success in many previous protein–ligand studies [40,41,42]. For half of the systems, IRDL can retrieve the top scoring solution with a RMSD below 2 or 3 Å when considering the backbone or the whole peptide, respectively. Results associated with the second half of the system are sometimes slightly above the limits of 2 and 3 Å, except for PDB ID 4RXH and 1OSZ systems, where the RMSD is high. Similar results are obtained with the SP-Peptide protocol.

Considering not only the top scoring docking solutions but also the rank of the first structure fulfilling the criteria for a correct docking solution, i.e., with a RMSD below 2 Å or 3 Å when considering the peptide backbone or the whole peptide, respectively, the results are slightly different (Table 1). 

It appears that IRDL protocol is able to retrieve a correct docking solution for each protein–peptide complex at the first or second positions for eight and ten protein–peptide complexes when considering the backbone or the whole peptide, respectively. However, for two complexes (PDB ID 4RXH and 3T6B) the best positions were at rank 36 and 67, respectively, considering the backbone only, but a correct solution is obtained at the first rank for PDB ID 3T6B when considering the whole peptide. Furthermore, those poses conserve most of the hydrogen bond interactions present in the crystal structure. As often observed in docking experiments, the missed hydrogen bonds are compensated by other hydrogen bonds present with close residues [41]. These observations assess the good efficiency of the IRDL protocol to retrieve the correct docking poses of the peptides and the conserved interactions observed in crystal structures. While SP protocol is able to correctly retrieve the binding mode of the opioid peptide Tynorphin (PDB ID 3T6B) at the first position, SP-peptide is, surprisingly, not able to retrieve any correct docking solution for this complex. For SP and SP-peptide, several complexes lead to docking issues. Four peptides are not docked correctly using the SP protocol (PDB ID 1OSZ, 3MMG, 4GRV, and 3CH8), and likewise for two peptides using the SP-peptide protocol (PDB ID 3T6B and 4RXH). For these six complexes, SP and SP-peptide protocols are not able to retrieve any docking solution with a RMSD below 2 Å or 3 Å, considering the peptide backbone or the whole peptide, respectively. However, one has to keep in mind that the SP protocol was not developed specifically for protein–peptide complexes. Furthermore, considering the results presented in Table 1, the IRDL method outperforms the two other protocols regarding the ranking of a correct docking solution. While the SP-Peptide can provide a correct solution for nine systems, the IRDL protocol always suggests a better rank of a correct solution regarding our RMSD criteria, considering either the backbone or the whole peptide, except for PDB ID 4Q6H, where IRDL retrieves the correct solution for the second docking solutions. It is important to note that both SP and SP-Peptide protocols fail in retrieving any docking solutions with low RMSDs, but a full minimization after docking could improve the results.

Despite the fact that we succeeded in demonstrating the efficiency and the originality of the IRDL method, in its current state, some limitations still exist. First, the position of the first fragment is a crucial step for building the peptide. Currently, the C-terminus is initially docked in the binding site, allowing building from the C-terminus to N-terminus only. Second, the first fragment must interact strongly with the receptor, since its position in the active site is critical for the success of peptide building. One improvement would be to dock all fragments and then grow them from the C- to N-terminus and N- to C-terminus sides. This approach is currently unfeasible because of long calculation times. Efforts are currently under way to improve IRDL.

### 2.2. Poses with the Lowest RMSD to the Crystal Structure

**Figure 2:**
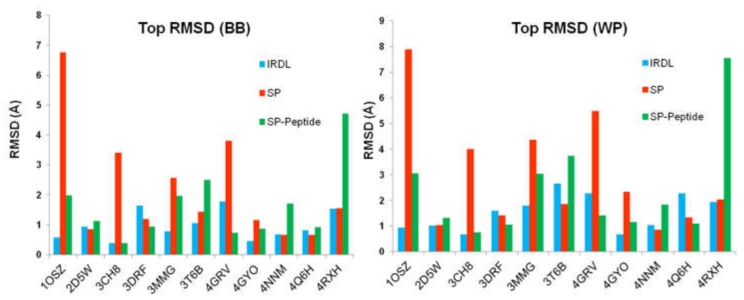
Pose with the lowest RMSD to the crystal structure for IRDL, SP, and SP-peptide protocols. BB: only the peptide backbone is considered; WP: the whole peptide is taken into account. RMSD values are given in Ångstrom.

In a second analysis, we checked if the IRDL protocol was able to retrieve the crystal structures of the eleven considered peptides, regardless of their ranking. Results, presented in Figure 2, show that in each case, IRDL was able to retrieve the crystal structures of the peptides bound to proteins with excellent precision. The docking pose with the lowest RMSD to the crystal structure was below 2 Å or 3 Å for each system, considering the backbone only or all atoms of the peptide, respectively. Remarkably, for some systems (PDB ID 1OSZ, 2D5W, 3CH8, 4GYO, and 4NNM), IRDL protocol was able to retrieve the top scoring pose with sub-angstrom deviation compared to the crystal structure. In comparison, considering the peptide backbone only, SP method was not able to retrieve a correct solution for four complexes, namely PDB ID 1OSZ, 3CH8, 3MMG, and 4GRV. Moreover, except for PDB ID 4NNM complex, SP protocol was not able to retrieve any docking solutions with sub-angstrom precision compared to the crystal structure, considering the entire peptide. Details of the best RMSD values are given in Table S2.

**Figure 3:**
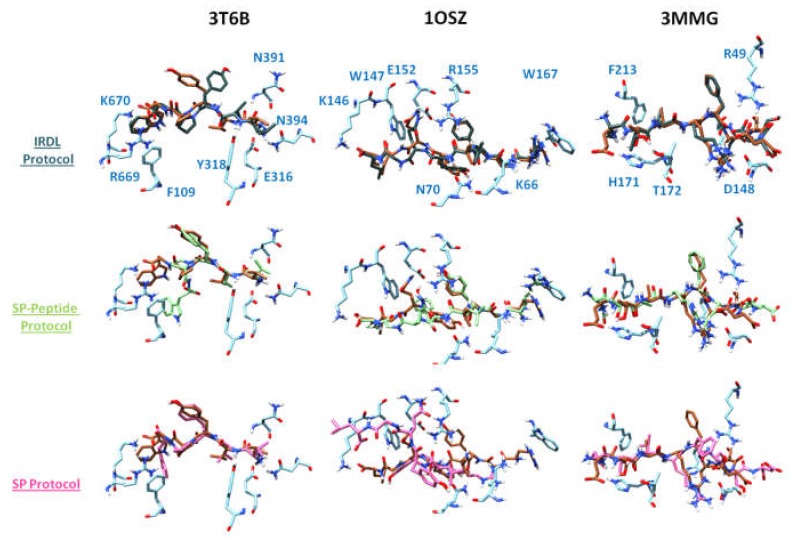
Poses with the lowest RMSD to the X-ray structure obtained with the three different protocols for PDB ID 3T6B, 1OSZ, and 3MMG complexes, respectively. X-ray positions of the peptides are represented in brown. Docking poses obtained with IRDL, SP-Peptide, and SP protocols are represented in dark blue, green, and pink, respectively.

Interestingly, SP-peptide protocol, which was developed specifically for the docking of peptide–protein complexes, did not outperform the two other methods in the prediction of protein–peptide interactions. For example, SP-Peptide was not able to find a satisfying docking pose for PDB ID 4RXH, the best pose with the lowest RMSD value, which presents a deviation equal to 4.7 Å and 7.5 Å compared to the crystal structure, considering the backbone and the whole peptide, respectively. In comparison, IRDL approach and SP protocol are able to retrieve excellent binding modes for the peptide for this complex with a similar RMSD of 1.5 and 2.0 Å for the backbone and whole peptide, respectively. Considering the PDB ID 1OSZ, 3MMG, and 3T6B, SP-peptide predicts the best poses of the peptides with a RMSD above 3 Å deviation from the crystal structure, considering the whole peptide (Figure 2). Regarding the IRDL approach, it appears that the results are always better than SP-peptide for those three cases (RMSD below 3 Å). However, results are better for SP-peptide when considering only the backbone (RMSD between 2.0 and 3.5 Å), but the values are still too high for accurate protein–peptide studies in drug design projects. Indeed, such deviations can bring about large errors in the orientation of the side chains, as shown in Figure 3 for PDB ID 3MMG. The RMSD of 2.0 Å obtained for PDB ID 3MMG is mainly due to the side chain of the last residues of the peptide. In the case of IRDL, the RMSD is only 0.8 Å and the last residues are better superimposed on the crystal structure. Surprisingly, for both complexes of PDB ID 4RXH and 3T6B, SP protocol gives better results for the pose with the lowest RMSD of the X-ray structure than SP-peptide, suggesting that these protocols are also protein–peptide complex dependent. For the other systems, we observe that IRDL protocol provides equivalent or better results (PDB ID 1OSZ) than those obtained with SP and SP-peptide protocols. The same observation could be made considering the whole peptide.

Figure 3 represents the superimposition of the crystallographic structures, with the docking poses having the lowest RMSD to the X-ray structure obtained for PDB ID 3T6B, 1OSZ, and 3MMG for the three different docking protocols. For PDB ID 3T6B complex, IRDL is able to predict the crystallographic binding mode of the opioid peptide Tynorphin with excellent precision (RMSD 1.1 Å) when considering the backbone. Regarding the whole peptide, it appears that the side-chain of the tyrosine residue is misplaced compared to the crystallographic position, where it interacts with F443 of human DPPIII. In this case, the phenol interacts with H450 in a buried part of the cavity, and makes a hydrogen bond with I390 (see Figure S1). The positions of the others residues are well superimposed with the residues of the crystal structure. SP method also suggests a correct solution (RMSD 1.4 Å), but it provides the wrong placement for the last tryptophan residue. The position of its side chain and the C-terminus are significantly shifted, pointing toward I672 (see Figure S1). Moreover, this residue seems to be critical for the interaction, since it forms two cation–π interactions with R669 and K670 of the receptor. SP-Peptide provides an alternative placement for the side chain, which interacts with R669 in a cation–π interaction, and a hydrophobic interaction with the phenyl ring of F109 of the protein. This alternative orientation of the tryptophan does not seem in the most favorable position, taking into account the interactions of residues in the crystal structure. On the contrary, SP protocol and particularly IRDL method correctly retrieve these specific interactions with R669 and K670.

Importantly, this tryptophan residue is included in the first fragment used by IRDL, meaning that this residue is in the first segment, docked with SP-peptide protocol. We hypothesize that the low number of rotatable bonds in the segment, compared to the whole peptide, allows SP-peptide to better dock this residue in the cavity and to find a conformation more similar to the crystal structure. Thus, it seems that in this particular case, splitting the whole peptide into segments induces a better exploration of the conformational space, and allows the docking algorithm to find the correct binding mode of the fragment more easily.

In the case of PDB ID 1OSZ, RMSD values for the pose with the lowest RMSD to the X-ray structure show that our method suggests an excellent solution that is very close to the X-ray structure of the 8-residue mutated vesicular stomatitis virus (VSV8 V4L) peptide. The deviation shows sub-angstrom accuracy regarding the backbone (RMSD of 0.6 Å) or the whole peptide (RMSD of 0.9 Å). For comparison, SP-peptide provides a reasonable docking solution of 1.9 and 3.1 Å for the backbone and the whole peptide, respectively, while SP method is not able to retrieve a solution below 6 Å. Regarding the structural details, IRDL is able to retrieve all major protein–peptide interactions present in the crystal structure (Figure 3). SP-Peptide protocol retrieves the atomic positions of the backbone with a good accuracy, but the orientation of the side chain of the docked tyrosine at the fifth position (Y5) is poorly predicted, since this residue interacts with E152 of class I MHC H-2 Kb instead of with hydrophobic residues V9 and V97. Consequently, Q6 residue of the peptide, which interacts with E152 in the crystal structure, is also mispredicted and points toward the bulk solvent. Regarding the docked solution obtained by SP, the peptide position is strongly deviated compared to the crystal structure.

The same observation can be made for the case of the PDB ID 3MMG complex, where the mutated Tobacco vein mottling virus (TVMV) is bound to canonical peptide substrates of 8 residues. IRDL protocol proposes the closest solution to the X-ray structure, with an RMSD of 0.8 Å considering the backbone. Only the C-terminus residue of the peptide is misplaced (E2), in which the position of the glutamate moiety is shifted, interacting with H171 of the protein instead of pointing toward the bulk solvent. SP-peptide also provides a correct docking solution, even if glutamine Q7 takes the position of arginine R5 in the crystallographic structure. The remaining part of the peptide is slightly shifted but the overall conformation is in good agreement with the crystallographic structure, with an RMSD of 2.6 Å. Concerning the SP method, despite a correct prediction of the position of the first residues, it appears that the residues F6 to D9 are located outside the protein cavity.

To summarize, this novel IRDL method is always able to retrieve a correct docking pose with RMSD below 2 Å or 3 Å compared to the crystal structure, considering the backbone or the whole peptide, respectively. Even if the top scoring pose is not always accurate (as also observed for SP and SP-Peptide), our protocol is able to retrieve a correct pose for all systems, which is not systematically the case for the two other algorithms. These results show the good efficiency of IRDL method in retrieving the most similar binding mode compared to crystal structures.

## 3. Materials and Methods

### 3.1. Datasets

Peptide–protein systems were downloaded from the PDB database (www.rcsb.org) [43]. Several criteria have been considered for system selection: only complexes containing peptides interacting with proteins are kept, resolved by X-ray crystallography method, and with a resolution below 3 Å. Structures have only natural residues in their sequence. Complexes in which the peptides are highly solvent-exposed and make numerous interactions with crystallographic water molecules were discarded in order to avoid additional complexity in evaluating IRDL. Visual inspection was performed to check the mode of interaction of the peptides. Finally, 11 structures were selected for this study (PDB ID: 1OSZ, 2D5W, 3CH8, 3DRF, 3MMG, 3T6B, 4GRV, 4GY0, 4NNM, 4Q6H, 4RXH). Details of these structures are presented in Table 2.

As mentioned in Table 2, the 11 peptide sequences contain between 5 and 8 amino acids, and between 15 (PDB ID 3T6B) and 43 (PDB ID 4RXH) torsion angles. Peptide sequences are detailed in the Supplementary Materials (Table S1). One can observe that different types of peptides were used, covering a diversity in flexibility and peptide types (charged, uncharged, hydrophobic).

### 3.2. Receptor Preparation

When multiple subunits were present, monomer A was selected in all cases. The 11 receptors were prepared with the Maestro protein preparation wizard using default parameters [44,45]. No side-chain or residue is missing on the protein in the direct neighborhood of all the studied peptides at 5 Å of the protein. OPLS-2005 force field was used in this study. Grids were generated with Glide by selecting the bound peptide to define the cavity, encompassing the entire binding sites in all the studied peptide–protein complexes. Physiological pH was used for residue protonation.

### 3.3. SP and SP-Peptide Docking

All peptides were prepared with the same protocol: a conformational search was performed using MacroModel [46] in order to propose different starting points for docking calculation. Default parameters for the conformational search (Mixed Torsional or Low-mode sampling algorithm) were considered. All conformations generated by MacroModel (up to 200 conformations) were used as starting points for docking. This first step was used to avoid the bias during docking introduced by using the crystallographic coordinates as the starting point. SP and SP-Peptide modules from Glide 6.5 [39] were used to perform the docking of the 11 ligands with default parameters. During docking, the receptor is considered fixed, while the peptide is kept fully flexible. 

### 3.4. Iterative Residue Docking and Linking Method

#### 3.4.1. File Preparation

**Figure 4:**
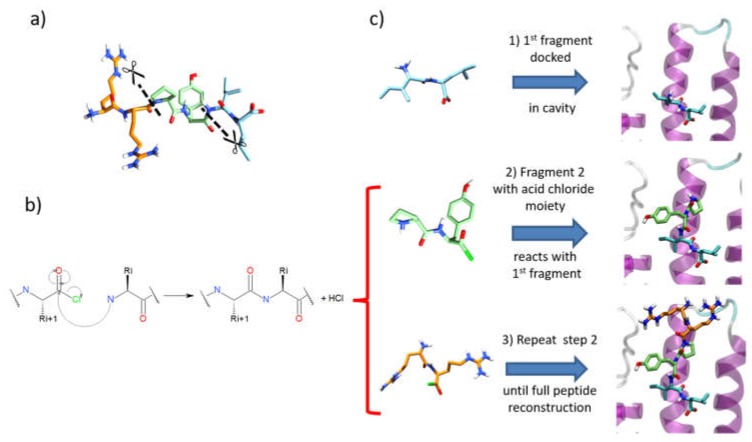
Summary of the IRDL (Iterative Residue Docking and Linking) protocol. (**a**) Fragmentation of the peptides following defined rules. (**b**) Scheme of the chemical reaction considered for the covalent docking module of Glide, and used in steps 2 and 3. (**c**) Peptide reconstruction with IRDL protocol: (**1**) Docking of the first segment in the receptor. (**2**) Intramolecular covalent docking with segment (2). (**3**) Step (2) is repeated with all successive fragments until the whole peptide is created.

We have developed a new methodology to dock peptides on proteins, using an intramolecular covalent docking approach. The objective is to split the whole peptides into several segments, to dock them individually and sequentially, and finally to link the segments together to form the original peptide using a covalent amide bond formation. Consequently, IRDL works in sequential steps (one step per segment), and this approach differs from classical docking, which treats the whole peptide at the same time. However, IRDL aims at solving the docking problem caused by the high degree of peptide flexibility. By fragmenting the peptides into a few segments, this approach reduces the number of rotatable bonds per molecule to dock. Therefore, the exploration of the conformational space for finding relevant conformers that can interact with the binding site is reduced. To reconstruct the peptide, segments were linked together by intramolecular covalent docking. In the covalent docking module of Glide [47], the calculation must be associated with a chemical reaction. A set of reactions is already included in the module, but the user can add new synthesis reactions in the database. Consequently, there is currently no limit on the reaction needed to perform the fragment growth. Here, we decided to choose a simple nucleophile addition/electrophile reaction, as shown in Figure 4, involving an acid chloride moiety on one of the fragments. This fragment (fragment *n* + 1) will react with the N-terminal amine of fragment *n* in order to create the peptide bond. This means that the reconstruction process of the peptide within the binding site goes from C-terminus to N-terminus. Regarding the fragmentation of the peptides into short segments, we set up several rules to optimize the procedure:

(a) Segments must have 10 or less rotatable bonds. This criterion allows simplification of each calculation by reducing flexibility, therefore improving the conformational search during docking.
(b) To avoid unfeasible covalent docking solutions, we considered that the N-terminal residue should not contain any nucleophilic sidechains. Thus, residues such as Tyr, Cys, Ser, Thr, Asn, and Gln were excluded as N-terminal residues in a fragment, since the covalent docking module of Glide considers those amino acids as nucleophilic moieties. Otherwise, when the molecular topology of the fragment is not conserved after intramolecular covalent docking, the solutions are removed during the post-process analysis (see below).

Once the peptides were fragmented, IRDL methodology was applied as follows:

(1) After a conformational search with MacroModel of the first fragment, as performed for SP and SP-Peptide docking, all conformers were docked in the binding site using the SP-Peptide module of Glide with default parameters. Then, we kept all docking poses up to the pose with the lowest RMSD to crystal structure. In a more realistic project, one would keep all docking poses, which will increase computational time but could improve the results.
(2) The second segment is docked using the covalent docking module of Glide (version 1.3) on each of the selected scoring poses of the first fragment. To allow the chemical reaction to occur, an acid chloride moiety is used on the C-terminal residue of the second fragment. During docking, this acid chloride moiety is automatically placed nearby the primary amine backbone of the first docked fragment. Glide will then optimize the interactions of the molecule in the binding site, keeping the distance between the acid chloride and the primary amine backbone. Then, in order to create the peptide bond, the chemical reaction between the N-terminal part of the first segment and the acid chloride moiety is made. Finally, both residues at the vicinity of the newly formed bond are optimized to remove strains and the receptor stays rigid. Then, the resulting poses are clustered into 15 clusters and a representative pose is proposed for each cluster. All cluster centers are ranked by the scoring function of the Glide covalent docking module. This scoring function averages the Glide docking score values of the two individual noncovalent poses and the resulting covalent poses involved in the peptide bond. Finally, all solutions are rescored with SP scoring function. At this step, we also evaluated the extra precision (XP) scoring function for those intermediate rescoring steps, but the results were less satisfactory (data not shown). As in step one, we decided to keep the highest scoring poses up to the pose with the lowest RMSD to the X-ray structure.
(3) Step 2 is repeated until the whole peptide is entirely created.

#### 3.4.2. Post-Process Analysis

All poses obtained from either classical docking or IRDL approach were compared to the crystallographic structure of the peptides. Analysis was performed by RMSD calculation between crystal structure and docked positions, considering only heavy atoms. For each linking step between two fragments, we kept all poses between the highest scoring pose (“best-scoring pose”) (top 1) and the pose with the lowest RMSD to the X-ray structure (“best-RMSD pose”), in order to increase the number of fragment poses to process. The ranking of all fragments for each peptide is analyzed to obtain the combination of the fragments associated with the best-RSMD pose and the best-scoring pose. It is important to note that the knowledge of the best-RMSD pose against the conformation of the crystal structure of the peptide is not compulsory in our approach. The main idea here is to validate the proof of concept of our methodology by considering a large number of poses with reasonable calculation times. A cut-off based on a scoring value could be applied at each step; as an example, it is possible to obtain a satisfying pose by considering the top 10 poses at each step (see Supplementary Materials, Table S3). The rescoring procedure is performed with the "score in place" method, in which the interaction energy is estimated by the Glide scoring function for the same conformation without modification of the coordinates of the peptide. Finally, all solutions obtained for all protocols were rescored with the XP scoring function, in order to improve the interaction score between the receptor and the peptide. Final scoring values were then compared between the three docking protocols.

## 4. Conclusions

Peptide–protein interactions represent more than a quarter of global interactions in cells. They are involved in numerous important processes, such as signaling, cell development, and migration. In this study, we proposed a novel approach to predict the binding mode of peptides to proteins. The innovative methodology, following specific rules, is based on splitting the peptides into several segments in order to reduce the conformational search space during docking, an important issue in docking algorithms. The generated fragments are then docked and linked from the C-terminal to the N-terminal. The first residue is docked using the SP-peptide protocol of Glide. Then, the covalent module of Glide is used to dock the next segment, and following defined atomic distances between the two fragments, an intramolecular covalent bond is created to form a peptide bond. A nucleophilic addition/elimination reaction between acyl chloride from the C-terminal of one fragment to the amine of the N-terminal of the adjacent fragment is performed, and the chemical reaction will create a peptide bond. This step is repeated until the peptide is reconstructed. The Iterative Fragment Docking and Linking (IRDL) approach has been validated on 11 different protein–peptide complexes. Even if the poses with lowest RMSD are not always scored as the top scoring position, we observe that the protocol is always able to retrieve a correct pose for all complexes with a RMSD below 2 or 3 Å compared to the crystal structures, considering only the backbone or the whole peptide, respectively. Regarding the performance of docking, IRDL provides similar results to the two docking protocols implemented in Glide (SP and SP-peptide). Each tool provides correct results for some specific complexes. In addition, errors on the position of either the backbone or the sidechain are more acceptable in the case of IRDL protocol, in contrast to the SP, and to a lesser extent, SP-peptide protocols that, in some cases, have a deviation higher than 5 Å, considering the pose with the lowest RMSD to the X-ray structure. We show that IRDL provides a reasonable accuracy in predicting the binding mode of a peptide on the protein surface.

The IRDL approach can be applied on any peptide–protein complexes harboring various binding sites, including a deep cavity or a solvent exposed surface. In addition, we provide here a proof of concept of the efficiency of the protocol to retrieve with good precision the correct structure of peptide–protein complexes. This new tool can already be used to design synthetic flexible peptide binding to a protein target. Nevertheless, some improvements will be implemented to the IRDL protocol in the future to optimize its performance and to make it fully automatic. Currently, the protocol provides a peptide construction from the C- to the N-terminus of the peptide using a classical chemical reaction. This is an actual limitation, especially in the case of peptides interacting in cavities by their N-terminus. To avoid this restriction, we envisage evaluation of further IRDL protocols by constructing the peptides from the N- to the C-terminus. Furthermore, a scoring function designed for protein–peptide interactions [48,49] could be implemented in order to obtain a better ranking of correct solutions. This new scoring function could take into account, for example, the particular behavior of peptides regarding flexibility, hydrophobic properties, cation–π interactions, etc. One can also envisage the use of Molecular Mechanics/Generalized-Born Surface Area (MM/GBSA) or Molecular Dynamics (MD)/GBSA for scoring the whole peptide. In addition, the induced fit mechanism could improve the performance of the docking tools, but this approach is often time consuming. 

We developed a novel and efficient approach to predict, with excellent accuracy compared to experimental data, the structure of protein–peptide complexes. This method could be useful to help in the design of new peptide therapeutics or peptidomimetics.

## Figures and Tables

**Table 1:** Rank of the first structure having a RMSD below 2 Å or 3 Å for the backbone or for the whole peptide considering the three protocols. BB: RMSD values considering the backbone; WP: RMSD values considering the whole peptide; “-“: no structure fulfilling the RMSD criteria; ^a^ ratio of the actual conserved hydrogen bonds between the crystal structure and the docked solution of the whole peptide (WP) obtained by IRDL.

PDB Code	IRDL			SP		SP-Peptide	
	BB	WP	H-Bond Number Ratio ^a^	BB	WP	BB	WP
1OSZ	2	2	1	-	-	12	2
2D5W	1	1	1	1	1	1	1
3CH8	1	1	1	-	-	1	1
3DRF	6	1	0.9	11	1	32	4
3MMG	1	1	1	-	-	3	2
3T6B	67	1	0.75	1	1	-	-
4GRV	1	1	0.5	-	-	1	1
4GYO	2	2	0.86	9	9	9	9
4NNM	1	1	0.82	1	1	27	1
4Q6H	2	2	0.88	1	1	1	1
4RXH	36	36	0.8	1	1	-	-

**Table 2:** Information of the 11 peptide–protein complexes used in the study.

PDB Code	Classification	Target	Resolution (Å)	Organism	Peptide Sequence Length	Torsion Angles
1OSZ	Complex (MHC-1)	H-2K1	2.1	*Mus Musculus*	8	33
2D5W	Protein Binding	Oligopeptide binding protein	1.3	*Thermus thermophilus*	5	18
3CH8	Protein Binding	PDZ-Fibronectin	1.9	*Homo Sapiens*	8	25
3DRF	Protein Binding	OppA	1.3	*Lactococcus lactis*	8	25
3MMG	Hydrolase	Tobacco Vein Mottling Virus protease	1.7	*Tobacco Vein Mottling Virus*	8	35
3T6B	Hydrolase	DPP III	2.4	*Homo Sapiens*	5	15
4GRV	Signaling protein	NTS1 receptor	2.8	*Rattus Norvegicus*	6	27
4GYO	Hydrolase	Rap-J	2.2	*Homo Sapiens*	5	22
4NNM	Protein Binding	TIP-1	1.6	*Homo Sapiens*	6	21
4Q6H	Transport Protein	CAL	1.9	*Homo Sapiens*	6	26
4RXH	Transport Protein	Importin-α	1.8	*Neurospora Crassa*	6	43

## Supplementary Materials

The following are available online. Table S1: Peptide and corresponding fragment sequences used in the study. Table S2: Top RMSD pose obtained from the three approaches. Table S3: Top scoring pose obtained for the three protocols after rescoring with XP. Figure S1: Detail of the top RMSD pose obtained with SP docking method and IRDL approach for PDB ID 3T6B.

## References

[1] Pawson T., Nash P. (2003). Assembly of Cell Regulatory Systems through Protein Interaction Domains. Science.

[2] Blobel G., Sabatini D.D., Manson L.A. (1971). Ribosome-Membrane Interaction in Eukaryotic Cells. Biomembranes: Volume 2.

[3] Petsalaki E., Russell R.B. (2008). Peptide-mediated interactions in biological systems: New discoveries and applications. Curr. Opin. Biotech..

[4] Tovar C., Rosinski J., Filipovic Z., Higgins B., Kolinsky K., Hilton H., Zhao X., Vu B.T., Qing W., Packman K. (2006). Small-molecule MDM2 antagonists reveal aberrant p53 signaling in cancer: Implications for therapy. Proc. Natl. Acad. Sci. USA.

[5] Perez M., Cuadros R., Benitez M.J., Jimenez J.S. (2004). Interaction of Alzheimer’s disease amyloid beta peptide fragment 25-35 with tau protein, and with a tau peptide containing the microtubule binding domain. J. Alzheimers Dis..

[6] Penna G., Amuchastegui S., Cossetti C., Aquilano F., Mariani R., Giarratana N., De Carli E., Fibbi B., Adorini L. (2007). Spontaneous and prostatic steroid binding protein peptide-induced autoimmune prostatitis in the nonobese diabetic mouse. J. Immunol..

[7] Sievers S.A., Karanicolas J., Chang H.W., Zhao A., Jiang L., Zirafi O., Stevens J.T., Munch J., Baker D., Eisenberg D. (2011). Structure-based design of non-natural amino-acid inhibitors of amyloid fibril formation. Nature.

[8] Illana G., Inna D. (2007). NAP, A Neuroprotective Drug Candidate in Clinical Trials, Stimulates Microtubule Assembly in the Living Cell. Curr. Alzheimer Res..

[9] Rahul J., Suresh C. (2005). Advancements in the Anti-Diabetes Chemotherapeutics Based on Amino Acids, Peptides, and Peptidomimetics. Mini RevMed. Chem..

[10] London N., Raveh B., Movshovitz-Attias D., Schueler-Furman O. (2010). Can self-inhibitory peptides be derived from the interfaces of globular protein–protein interactions?. Proteins Struct. Funct. Bioinform..

[11] Murray J.K., Gellman S.H. (2007). Targeting protein–protein interactions: Lessons from p53/MDM2. Pept. Sci.

[12] Minkovsky N., Berezov A. (2008). BIBW-2992, a dual receptor tyrosine kinase inhibitor for the treatment of solid tumors. Curr. Opin. Investig. Drugs.

[13] Filippo M., Yi Z. (2009). Targeting Rho GTPases by Peptidic Structures. Curr. Pharm. Des..

[14] Vlieghe P., Lisowski V., Martinez J., Khrestchatisky M. (2010). Synthetic therapeutic peptides: Science and market. Drug Discov. Today.

[15] Milhas S., Raux B., Betzi S., Derviaux C., Roche P., Restouin A., Basse M.-J., Rebuffet E., Lugari A., Badol M. (2016). Protein–Protein Interaction Inhibition (2P2I)-Oriented Chemical Library Accelerates Hit Discovery. ACS Chem. Biol..

[16] Arkin M.R., Whitty A. (2009). The road less traveled: Modulating signal transduction enzymes by inhibiting their protein-protein interactions. Curr. Opin. Chem. Biol..

[17] London N., Raveh B., Schueler-Furman O. (2012). Modeling Peptide-Protein Interactions.

[18] London N., Raveh B., Schueler-Furman O. (2013). Peptide docking and structure-based characterization of peptide binding: From knowledge to know-how. Curr. Opin. Struct. Biol..

[19] Wu G., Chen Y.-G., Ozdamar B., Gyuricza C.A., Chong P.A., Wrana J.L., Massagué J., Shi Y. (2000). Structural Basis of Smad2 Recognition by the Smad Anchor for Receptor Activation. Science.

[20] Rubinstein M., Niv M.Y. (2009). Peptidic modulators of protein-protein interactions: Progress and challenges in computational design. Biopolymers.

[21] Schindler C.E.M., de Vries S.J., Zacharias M. (2015). Fully Blind Peptide-Protein Docking with pepATTRACT. Structure.

[22] Lamiable A., Thévenet P., Rey J., Vavrusa M., Derreumaux P., Tufféry P. (2016). PEP-FOLD3: Faster de novo structure prediction for linear peptides in solution and in complex. Nuc. Acids Res..

[23] Raveh B., London N., Zimmerman L., Schueler-Furman O. (2011). Rosetta FlexPepDock ab-initio: Simultaneous Folding, Docking and Refinement of Peptides onto Their Receptors. PLoS ONE.

[24] Trellet M., Melquiond A.S.J., Bonvin A.M.J.J. (2013). A Unified Conformational Selection and Induced Fit Approach to Protein-Peptide Docking. PLoS ONE.

[25] Tubert-Brohman I., Sherman W., Repasky M., Beuming T. (2013). Improved Docking of Polypeptides with Glide. J. Chem. Inf. Model.

[26] Antes I. (2010). DynaDock: A new molecular dynamics-based algorithm for protein–peptide docking including receptor flexibility. Proteins Struct. Funct. Bioinform..

[27] Blaszczyk M., Kurcinski M., Kouza M., Wieteska L., Debinski A., Kolinski A., Kmiecik S. (2016). Modeling of protein–peptide interactions using the CABS-dock web server for binding site search and flexible docking. Methods.

[28] London N., Movshovitz-Attias D., Schueler-Furman O. (2010). The Structural Basis of Peptide-Protein Binding Strategies. Structure.

[29] Böhm H.-J. (1993). A novel computational tool for automated structure-based drug design. J. Mol. Recognit..

[30] Miranker A., Karplus M. (1995). An automated method for dynamic ligand design. Proteins Struct. Funct. Bioinform..

[31] Durrant J.D., Amaro R.E., McCammon J.A. (2009). AutoGrow: A Novel Algorithm for Protein Inhibitor Design. Chem. Biol. Drug Des..

[32] Durrant J.D., Lindert S., McCammon J.A. (2013). AutoGrow 3.0: An improved algorithm for chemically tractable, semi-automated protein inhibitor design. J. Mol. Graph. Model..

[33] Welch W., Ruppert J., Jain A.N. (1996). Hammerhead: Fast, fully automated docking of flexible ligands to protein binding sites. Chem. Biol..

[34] Kramer B., Rarey M., Lengauer T. (1999). Evaluation of the FLEXX incremental construction algorithm for protein-ligand docking. Proteins.

[35] Moon J.B., Howe W.J. (1991). Computer design of bioactive molecules: A method for receptor-based de novo ligand design. Proteins Struct. Funct. Bioinform..

[36] Sawyer T.K., Fisher J.F., Hester J.B., Smith C.W., Tomasselli A.G., Tarpley W.G., Burton P.S., Hui J.O., McQuade T.J., Conradi R.A. (1993). Peptidomimetic inhibitors of human immunodeficiency virus protease (HIV-PR): Design, enzyme binding and selectivity, antiviral efficacy, and cell permeability properties. Bioorg. Med. Chem. Lett..

[37] Thanki N., Rao J.K.M., Foundling S.I., Wlodawer A., Howe W.J., Moon J.B., Hui J.O., Tomasselli A.G., Heinrikson R.L., Thaisrivongs S. (1992). Crystal structure of a complex of HIV-1 protease with a dihydroxyethylene-containing inhibitor: Comparisons with molecular modeling. Protein Sci..

[38] Thaisrivongs S., Tomasselli A.G., Moon J.B., Hui J., McQuade T.J., Turner S.R., Strohbach J.W., Howe W.J., Tarpley W.G., Heinrikson R.L. (1991). Inhibitors of the protease from human immunodeficiency virus: Design and modeling of a compound containing a dihydroxyethylene isostere insert with high binding affinity and effective antiviral activity. J. Med. Chem..

[39] (2014). Glide.

[40] Bissantz C., Folkers G., Rognan D. (2000). Protein-Based Virtual Screening of Chemical Databases. 1. Evaluation of Different Docking/Scoring Combinations. J. Med. Chem..

[41] Kellenberger E., Rodrigo J., Muller P., Rognan D. (2004). Comparative evaluation of eight docking tools for docking and virtual screening accuracy. Proteins Struct. Funct. Bioinform..

[42] Cummings M.D., DesJarlais R.L., Gibbs A.C., Mohan V., Jaeger E.P. (2005). Comparison of Automated Docking Programs as Virtual Screening Tools. J. Med. Chem..

[43] Berman H.M., Westbrook J., Feng Z., Gilliland G., Bhat T.N., Weissig H., Shindyalov I.N., Bourne P.E. (2000). The Protein Data Bank. Nuc. Acids Res..

[44] (2014). Maestro.

[45] Madhavi Sastry G., Adzhigirey M., Day T., Annabhimoju R., Sherman W. (2013). Protein and ligand preparation: Parameters, protocols, and influence on virtual screening enrichments. J. Comput. Aid Mol. Des..

[46] (2014). MacroModel.

[47] Zhu K., Borrelli K.W., Greenwood J.R., Day T., Abel R., Farid R.S., Harder E. (2014). Docking Covalent Inhibitors: A Parameter Free Approach To Pose Prediction and Scoring. J. Chem. Inf. Model.

[48] Frank A.M. (2009). A Ranking-Based Scoring Function for Peptide–Spectrum Matches. J. Proteome Res..

[49] Spiliotopoulos D., Kastritis P.L., Melquiond A.S.J., Bonvin A.M.J.J., Musco G., Rocchia W., Spitaleri A. (2016). dMM-PBSA: A New HADDOCK Scoring Function for Protein-Peptide Docking. Front. Mol. Biosci..

