# Supplementary material for: In Silico Peptide Ligation: Iterative Residue Docking and Linking as a New Approach to Predict Protein-Peptide Interactions
Source: Molecules. 2019 Apr 5;24(7):1351. doi: 10.3390/molecules24071351 (PMC6480567; doi:10.3390/molecules24071351)
Supplement: Supplementary file 1 [file molecules-24-01351-s001.pdf]

Supplementary Materials

In Silico Peptide Ligation: Iterative Residue Docking and Linking as a New Approach to Predict Protein-Peptide Interactions

Julien Diharce[1], Mickaël Cueto[1], Massimiliano Beltramo[2], Vincent Aucagne[3], and Pascal Bonnet[1*]

1 Institut de Chimie Organique et Analytique (ICOA), UMR CNRS-Université d'Orléans 7311, Université d'Orléans BP 6759, 45067, Orléans Cedex 2, France
2 UMR Physiologie de la Reproduction et des Comportements (INRA, UMR85; CNRS, UMR7247; Université de Tours; IFCE), F-37380 Nouzilly, France
3 Centre de Biophysique Moléculaire (CNRS UPR4301), Rue Charles Sadron, F-45071 Orléans cedex 2, France

* Correspondence: pascal.bonnet@univ-orleans.fr; Tel.: +33-238-417-254

Table S1: Peptide and corresponding fragment sequences used in the study.

PDB ID	Peptide sequence	Segments obtained for IRDL protocol
1OSZ	RGYLYQGL	RGY<br>LYQ<br>GL
2D5W	KPKSA	KPK<br>SA
3CH8	PQPVDSWV	PQ<br>PV<br>DS<br>WV
3DRF	GSAISNSA	GS<br>AI<br>SN<br>SA
3MMG	GTVRFQSD	GTV<br>RF<br>QSD
3T6B	VVYPW	VV<br>YPW
4GRV	RRPYIL	RR<br>PY<br>IL

4GYO	ERMGT	ER<br>MGT
4NNM	YPTSII	YP<br>TS<br>II
4Q6H	LRTEQV	LRT<br>EQV
4RXH	PPKKKRKV	PPK<br>KK<br>RKV

Table S2: Top RMSD pose obtained from the three approaches.

PDB ID	Top RMSD pose						
	Iterative Covalent Docking (IRDL)			SP protocol		SP-Peptide protocol	
	RMSD (Å)		Segment ranking for each step	RMSD (Å)		RMSD (Å)	
	BB	WP		BB	WP	BB	WP
1OSZ	0.6	0.9	3, 1, 5	6.8	7.9	2.0	3.1
2D5W	0.9	1.0	1, 1	0.9	1.0	1.1	1.3
3CH8	0.4	0.7	37, 1, 9, 2	3.4	4.0	0.4	0.8
3DRF	1.6	1.6	7, 1, 6	1.2	1.0	1.0	0.9
3MMG	0.8	1.8	1, 2, 2	2.6	4.4	2.0	3.0
3T6B	1.0	2.7	39, 5	1.4	1.9	3.5	3.7
4GRV	1.8	2.3	1, 3, 4	3.8	5.5	0.7	1.4
4GYO	0.5	0.7	2, 1	1.2	2.3	0.9	1.2
4NNM	0.7	1.0	131, 1, 1	0.7	0.9	1.7	1.8
4Q6H	0.8	2.3	39, 9	0.7	1.3	0.9	1.1
4RXH	1.5	1.9	90, 1, 1	1.6	2.0	4.7	7.6

Table S3: Top scoring pose obtained for the three protocols after rescoring with XP.

PDB ID	Top Scoring pose						
	IRDL			SP protocol		SP-Peptide protocol	
	RMSD (Å)		Segment ranking for each step	RMSD (Å)		RMSD (Å)	
	BB	WP		BB	WP	BB	WP
1OSZ	4.2	6.1	4, 8, 10	8.6	10.4	5.7	4.4
2D5W	1.2	1.3	2, 1	1.6	1.5	1.3	1.4
3CH8	2.0	2.9	31, 1, 2, 3	5.2	5.6	1.1	0.9
3DRF	2.7	2.9	1, 1, 11	2.7	2.3	3.7	3.0
3MMG	0.8	1.8	1, 2, 1	10.8	10.8	2.1	4.2
3T6B	2.7	2.9	1, 1	1.5	1.9	2.8	5.0
4GRV	1.8	2.3	1, 4, 8	2.3	6.7	1.1	2.8
4GYO	3.2	3.9	1, 3	3.1	3.5	3.1	3.5
4NNM	0.9	1.6	131, 1, 11	0.8	1.0	2.7	2.4
4Q6H	3.3	3.9	3, 3	1.0	2.4	1.1	1.3
4RXH	4.4	7.0	40, 3, 6	1.5	2.0	7.1	8.8

The segment combination is associated to the ranking of each segment at each step of the IRDL protocol. It appears that the final combination associated to the lowest RMSD to the X-ray structure poses is not associated each time to the lowest RMSD segment position at each step. The same observation can be made for the best scoring pose. The top scoring segment at each step will not lead necessarily to the top scoring pose once the peptide reconstruction is complete. Nevertheless, it appears that a satisfying pose is found in 75% of the cases by considering the top 10 poses obtained at each step.

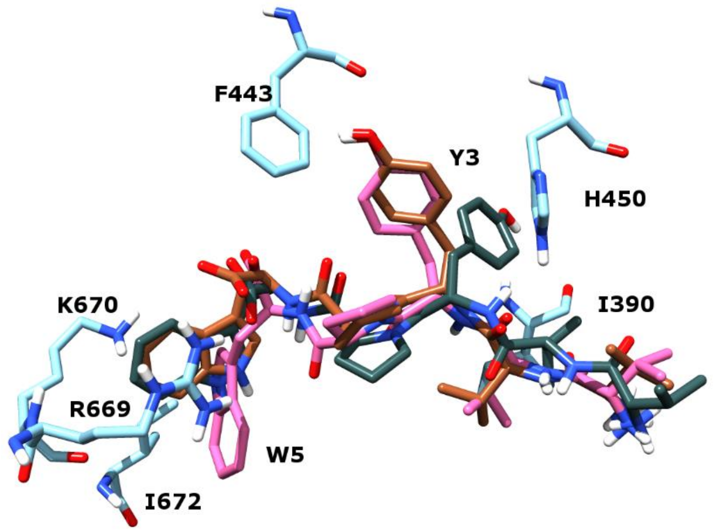

<u>Fig S1:</u> Detail of the top RMSD pose obtained with SP docking method and IRDL approach for PDB ID 3T6B. For the SP solution, the W5 residue does not interact anymore with K670 and R669, and points toward I672. Regarding the IRDL pose, the Y3 residue does not interact with F443 but with H450 and an H-bond with I390. Pink: SP solution; Dark blue: IRDL solution; Brown: crystallographic structure of the peptide.
